# 1349. Unveiling the Link between COVID-19 and the Gut Microbiome in an Outpatient Cohort with Mild to Moderate Illness

**DOI:** 10.1093/ofid/ofad500.1186

**Published:** 2023-11-27

**Authors:** Isin Y Comba, Ruben A Mars, Mitchell Dumais, Trena Van Gorp, Jonathan Harrington, John C O’Horo, Purna C Kashyap

**Affiliations:** Division of Public Health, Infectious Diseases, and Occupational Medicine, Department of Medicine, Mayo Clinic, Rochester, Minnesota; Mayo Clinic, Rochester, Minnesota; Mayo Clinic Rochester, Rochester, Minnesota; Mayo Clinic, Rochester, Minnesota; Mayo Clinic, Rochester, Minnesota; Mayo Clinic, Rochester, Minnesota; Mayo Clinic, Rochester, Minnesota

## Abstract

**Background:**

As the acute phase of the COVID-19 pandemic subsides, an increasing number of individuals are recognized as having long COVID, affecting over 65 million people worldwide. Recent studies have suggested a potential link between long COVID and alterations in the gut microbiome. In this study, we examined the gut microbiome of a large outpatient cohort during acute phase of infection, with the ultimate goal of determining predictors of long-term COVID-19 health outcomes.

**Methods:**

We conducted a study of 394 COVID-19 patients with positive SARS-CoV-2 testing from November 2020 to September 2021 at a multi-site healthcare system. Long COVID was defined as the persistence of symptoms for four weeks or more after illness onset. DNA extraction was performed using the MoBio PowerSoil Pro DNA isolation kit. Samples were sequenced using shotgun metagenomics. Stool SARS-CoV-2 RNA was tested using qRT-PCR of the E gene, and stool ELISA was performed for calprotectin levels. Standard biostatistical tools in R (v 4.2.3) were used for statistical analysis.

**Results:**

Of the 394 COVID-19 patients, 84 (21%) had long COVID, with fatigue being the most common persistent symptom (Table 1). No significant differences were seen in age, BMI, and antibiotic use. Those with long COVID were more likely to be female, had higher Charlson's comorbidity scores, more severe index illnesses. However, the two groups had no difference in SARS-CoV-2 stool PCR positivity or stool calprotectin levels (Table 2). Principal coordinate analysis of Bray Curtis dissimilarities revealed that patients with long COVID had a distinct microbial composition compared with those without long COVID during the acute phase of infection (ANOSIM R2= 0.005, P= 0.01). Categorizing patients by number of long COVID symptoms showed significant differences among groups (ANOSIM R2=0.1, P=0.007), mainly between patients with >3 symptoms and those with 0-1 symptoms (Bonferroni, P= 0.018). Alpha diversity was lower in patients with > 3 symptoms, although this was not statistically significant (Shannon diversity, two-way-ANOVA, P=0.051).

**Figure:**
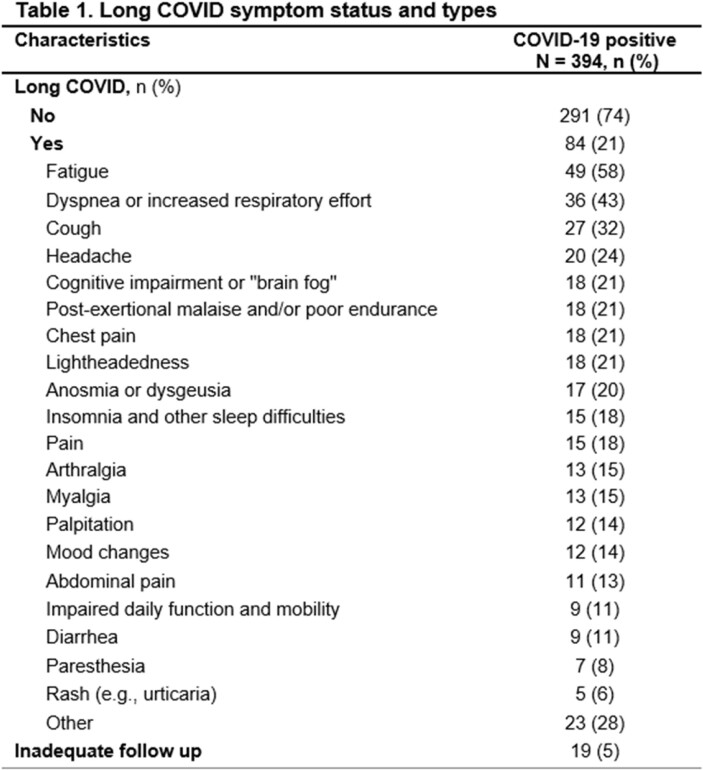

**Figure:**
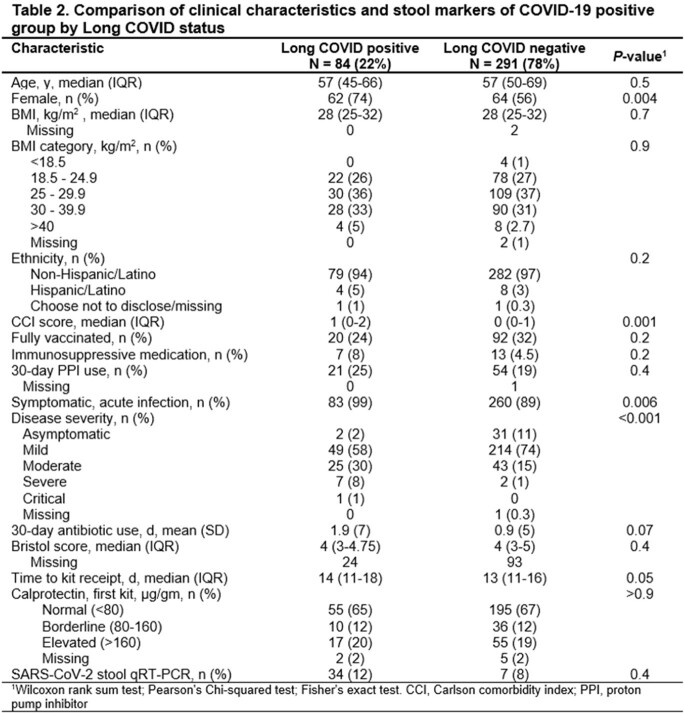

**Figure 1**

**Figure:**
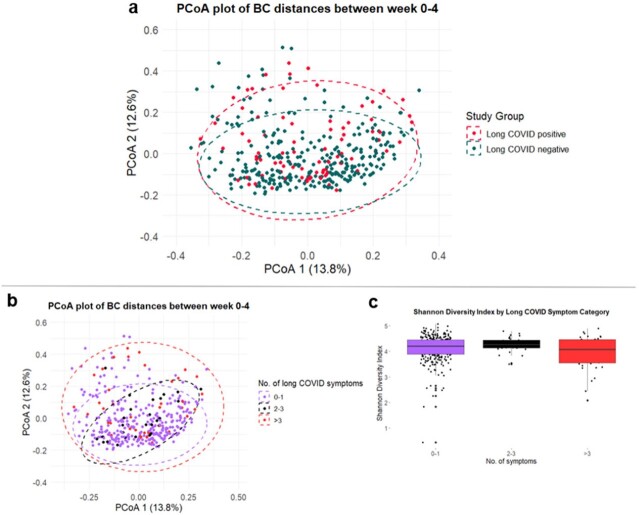

**Conclusion:**

Long COVID patients show distinct gut microbial composition during the acute phase of infection, suggesting a potential role in predicting long-term COVID health outcomes.

**Disclosures:**

**John C. O'Horo, Sr., MD, MPH**, Janssen Pharmaceuticals.: Grant/Research Support|nference: Grant/Research Support **Purna C. Kashyap, MD**, Intrinsic Medicine: Advisor/Consultant|Novome Biotechnologies: Advisor/Consultant|Pendulum Therapeutics: Advisor/Consultant

